# Effect of Comprehensive Cerebral Protection Program on Cerebral Oxygen Metabolism and Vascular Endothelial Function in Elderly Patients with Acute Cerebral Infarction

**Published:** 2019-02

**Authors:** Meihong ZHOU, Zhaojun HUANG

**Affiliations:** Department of Neurology, The First Affiliated Hospital of Nanchang University, Nanchang 330000, P.R. China

**Keywords:** Acute cerebral infarction, Senile, Comprehensive cerebral protection, Cerebral oxygen metabolism, Endothelial function

## Abstract

**Background:**

We aimed to explore the effect of comprehensive cerebral protection on cerebral oxygen metabolism and vascular endothelial function in elderly patients with acute cerebral infarction.

**Methods:**

A total of 168 elderly patients with acute cerebral infarction treated in The First Affiliated Hospital of Nanchang University, China from January 2016 to January 2018 were selected. The patients were divided into a control group and an observation group using random number method, n=84. Patients in the observation group were given comprehensive cerebral protection treatment, and patients in the control group were treated with conventional standardized treatments. The changes of cerebral oxygen metabolism, hemorheology and vascular endothelial function before and after treatment were compared between the two groups.

**Results:**

After treatment, oxygen content in arteries and internal jugular veins (Da-vO_2_), ofoxygen uptake fraction (OEF), Oxygen saturation (SpO_2_), nitric oxide (NO) were increased in both groups in comparison to before treatment, jugular venous oxygen saturation (SjvO_2_), brain oxygen uptake rate (ERO_2_), endothelin (ET), intracranial pressure (ICP), whole blood viscosity, plasma viscosity, reduced viscosity of whole blood, and hematocrit were decreased. However, the changes in the observation group were larger than those in the control group, the difference was statistically significant (*P*<0.05).

**Conclusion:**

The treatment of cerebral infarction in elderly patients with acute cerebral infarction can effectively improve the cerebral oxygen metabolism and vascular endothelial function and improve the blood rheology, which has important clinical value.

## Introduction

Cerebral infarction is caused by cerebral blood supply disorders of hypoxia, ischemia, resulting in local cerebral ischemic necrosis or brain softening (1). In patients with acute cerebral infarction, there is an ischemic and semi-shaded area around the infarcted lesion. Brain tissue hypoxia, ischemia, and metabolic disorders not only directly participate in the process of brain injury, but also work together with other secondary injury mechanisms to aggravate brain tissue damage. (2). And NO, ET and ICP also play an important biological activity in acute cerebral ischemia, and participate in the occurrence and development of cerebral infarction.

Therefore, it is of great significance to improve the collateral circulation and blood vessel function in clinical treatment, supply enough blood, rescue the reversibly damaged neurons, and reduce the incidence of reperfusion injury (3).

In this study, patients with long-term hypothermia treatment, ventilator hyperoxia treatment, early tracheotomy, sedative analgesia, early enteral nutrition, internal environment and intracranial pressure stabilization were treated with comprehensive cerebral protection in order to improve Cerebral oxygen metabolism, vascular endothelial function, and hemorheology in elderly patients with acute cerebral infarction to improve prognosis (4).

## Materials and Methods

### General Information

The 168 elderly patients with acute cerebral infarction treated in The First Affiliated Hospital of Nanchang University, China from January 2016 to January 2018 were enrolled as the study subjects. The patients were divided into a control group and an observation group by random number method, n=84. There were 51 males and 33 females in the observation group. The age ranged from 65 to 87 yr old and averaged (72.19±4.92) yr old. Onset to treatment time was 0.5–4.2 h with an average of (2.41±1.22) hours. Infarct sites: 38 cases of basal ganglia, 23 cases of lobar, 17 cases of thalamus, and 6 cases of other. There were 47 males and 37 females in the control group. The age ranged from 65 to 88 and the average age was (73.03±4.65) years. Onset to treatment time was 0.5–4.5 h, with an average of 2.51±1.31 hours. Infarct sites: 39 basal ganglia sites, 21 brain lobe sites, 19 thalamus, and 5 others. There was no significant difference in the general data between the two groups (*P*>0.05) and the data were comparable.

Inclusion criteria: 1) Patients with clinically diagnosed acute cerebral infarction; 2) Patients aged ≥ 65 years; 3) family members who voluntarily participated in this study and have signed informed consent.

Exclusion criteria: 1)mixed stroke patients; 2)combined heart, liver, kidney, lung, hematopoietic system and other functional disorders; 3) ipsilateral lesion history and sequelae; 4) irregular sleepers or mental illness patients.

### Methods

Patients in the observation group were given comprehensive cerebral protection treatment, and patients in the control group were treated with conventional standardized treatments.

### Control group

Conventional standardized treatment was adopted, including ventilator-assisted breathing, protection of brain cells, anti-inflammatory, lowering intracranial pressure, anti-infection, maintaining internal stability, nutrition, nerves and other standard treatment.

### Observation group

Comprehensive brain protection treatment was used. 1)Ventilator hyperoxia treatment: a ventilator was used to increase the concentration of oxygen in the inhaled gas (100% pure oxygen) and positive end expiratory pressure (8 cmH_2_O-10 cmH_2_O), continue treatment for 1h, once every 8h, 10 times were a course of treatment; 2)long-term mild hypothermia: right femoral vein puncture into the Cool Line catheter, connected with the intravascular heat exchange system, the target temperature was set to 34 °C, maintained until 5 days after operation, according to 0.25 °C / h rewarming, When the temperature reaches 36 °C, the intravascular heat exchange system was evacuated and naturally rewarmed. 3)Sedation and analgesia: propofol 0.5 mg/(kg·h)-3.0 mg/(kg·h) and fentanyl 25 μg/h-200 μg/h intravenously to maintain bispectral index of brain at 60–80; 4)early tracheotomy: percutaneous tracheotomy within 24 hours; 5) internal environment stability: hematocrit ≥ 0.36, blood Na^+^130mmol/L-150mmol/ L; 6)enteral nutrition: enteral feeding within 24h, 3d–5d to achieve adequate feeding; 7)intracranial pressure: if ICP ≥ 20 mmHg, intravenous infusion of 150ml of 20% mannitol, interval 6h, for patients with high intracranial pressure, cerebrospinal fluid was considered.

### Observation indicators

The changes in cerebral oxygen metabolism, hemorheology, and vascular endothelial function before and after treatment were compared between the two groups of patients. 1) Cerebral oxygen metabolism: including Da-vO_2_, OEF, SjvO_2_, ERO_2_, and SpO_2_, 4 ml of radial arterial blood was collected, 4 ml of blood of jugular vein was obtained, blood gas analysis was performed by blood gas analyzer, and various oxygen metabolism indexes were calculated; 2)Hemorheology: Determination and calculation of whole blood viscosity, plasma viscosity, reduced viscosity of whole blood, and hematocrit by capillary viscometer assay; 3) Vascular endothelial function: Transcranial Doppler flow analyzer was used to monitor changes in ICP, ET levels were measured by radioimmunoassay and NO levels were measured by chemiluminescence.

### Statistical methods

SPSS20.0 software (Chicago, IL, USA) was used for statistical analysis, measurement data were analyzed using *t* test, *P*<0.05 for the difference was statistically significant.

## Results

### Changes in cerebral oxygen metabolism index before and after treatment

There were no significant differences in Da-vO_2_, OEF, SjvO_2_, ERO_2_, and SpO_2_ between the two groups before treatment; Da-vO_2_, OEF and SpO_2_ was higher after treatment than before treatment in the two groups of patients, SjvO_2_, ERO_2_ were decreased in comparison to before treatment, but the changes in patients in the observation group were greater than those in the control group, the difference was statistically significant (*P*<0.05) (Table 1).

**Table 1:** Comparison of changes in cerebral oxygen metabolism before and after treatment

***Group***	***Time***	***Da-vO_2_ (ml/L)***	***OEF (%)***	***SjvO_2_ (%)***	***ERO_2_ (%)***	***SpO_2_(%)***
Observation group (n=84)	Before treatment	34.38±4.82	23.98±4.06	78.40±5.46	36.19±5.13	92.21±2.11
	After treatment	46.90±5.02	32.27±4.17	61.10±4.72	19.33±4.78	99.90±0.55
*t*		1.975	2.509	2.352	2.793	2.569
*P*		0.049	0.021	0.034	0.007	0.012
Control group (n=84)	Before treatment	34.20±4.71	23.89±5.18	78.51±5.22	36.29±4.83	92.20±2.41
	After treatment	42.19±4.55^*^	29.78±4.17^*^	66.83±5.11^*^	24.27±5.10^*^	96.43±0.91^*^
*t*		2.559	2.557	2.147	2.479	2.396
*P*		0.014	0.015	0.040	0.027	0.031

Note:

* indicates that there is a statistically significant difference between the control group and the observation group (*P*<0.05)

### Changes of hemorheology before and after treatment

Comparison of blood viscosity, plasma viscosity, reduced blood viscosity and hematocrit of whole blood before and after treatment showed no significant difference (*P*>0.01); Blood viscosity, plasma viscosity, reduced viscosity of whole blood and hematocrit were all lower than those before treatment, but the changes in patients in the observation group were greater than those in the control group, and the difference was statistically significant (*P*<0.01) (Table 2).

**Table 2:** Comparison of Changes of Hemorheology Before and After Treatment

***Group***	***Time***	***Whole blood viscosity (mPa·s)***			***Plasma viscosity (mPa·s)***	***Reduced viscosity of whole blood (mPa·s)***			***Hematocrit (%)***

		***Low shear rate***	***Medium shear rate***	***High shear rate***		***Low shear rate***	***Medium shear rate***	***High shear rate***	
Observation group (n=84)	Before treatment	16.49±2.19	9.03±1.22	7.29±1.30	3.53±0.48	27.19±3.32	13.17±2.20	9.55±1.61	56.13±2.75
	After treatment	10.33±2.04	5.26±1.10	3.57±1.07	1.65±0.41	17.36±3.21	8.56±2.05	6.53±1.48	43.48±2.66
*t*		1.996	2.492	2.561	2.352	2.678	2.147	2.767	2.559
*P*		0.047	0.024	0.013	0.034	0.009	0.040	0.008	0.014
Control group (n=84)	Before treatment	16.54±2.31	9.01±1.17	7.32±1.31	3.51±0.51	27.25±3.13	13.13±2.31	9.53±1.72	56.17±2.45
	After treatment	12.58±2.21^*^	7.08±1.10^*^	5.04±0.99^*^	2.28±0.55^*^	20.58±3.31^*^	10.72±2.23^*^	7.62±1.50^*^	47.40±2.90^*^
*t*		2.801	2.317	2.396	2.253	2.549	1.996	2.537	2.511
*P*		0.006	0.035	0.031	0.038	0.016	0.047	0.017	0.020

Note:

* indicates that there is a statistically significant difference between the control group and the observation group (*P*<0.05)

### Changes in vascular endothelial function before and after treatment

There was no significant difference in ET, ICP, and NO between the two groups before treatment.

After treatment, ET and ICP were lower in both groups than before treatment, and NO increased, but the changes in the observation group were larger than those in the control group, and the difference was statistically significant (*P*<0.01) (Table 3).

**Table 3:** Changes in vascular endothelial function before and after treatment

***Group***	***Time***	***ET (ng/L)***	***ICP (mmHg)***	***NO (μmol/L)***
Observation group (n=84)	Before treatment	74.66±7.18	15.87±7.21	62.19±22.17
	After treatment	51.04±6.13	11.21±3.34	104.29±27.84
*t*		2.517	2.435	2.037
*P*		0.019	0.029	0.044
Control group (n=84)	Before treatment	73.57±7.72	16.08±6.41	65.47±26.83
	After treatment	60.22±5.89^*^	14.32±3.17^*^	82.25±27.18^*^
*t*		2.524	3.098	2.377
*P*		0.018	0.002	0.033

Note:

* indicates that there is a statistically significant difference between the control group and the observation group (*P*<0.05)

## Discussion

The lesions in patients with acute cerebral infarction consist of a central necrotic zone and the surrounding ischemic penumbra. Since the brain cells in the necrotic zone are completely ischemic and their brain cells die, there is still collateral circulation in the penumbra of the ischemic region and a certain blood supply can be obtained. In addition, there are still a large number of viable neurons, the rapid recovery of blood can effectively improve brain metabolism, brain damage is still reversible, and nerve cells can still survive and restore normal function (5). Therefore, the protection of reversibly damaged neurons in clinical is a key point for the treatment of patients with acute cerebral infarction, by improving the function of the collateral circulation blood vessels around the ischemic region, ensuring oxygen supply and blood supply, and promoting the function of surviving neurons in the penumbra, better clinical efficacy will be achieved (6). Ventilator hyperoxia treatment maintains the patient at a certain oxygen concentration and pressure, uses α-adrenergic-like actions to constrict blood vessels, reduce local blood volume, eliminate cerebral edema, therefore achieve therapeutic goals (7). Hypothermia therapy reduces body temperature to 28°C–35°C by chemical or physical methods, inhibits the release of harmful substances such as NO, free radicals, and excitatory amino acids, relieves inflammation, protects the blood-brain barrier, and reduces brain metabolism. In turn, it exerts a protective effect on human tissues (8). In this study, the elderly patients with acute cerebral infarction were treated with a comprehensive brain protection program combined with multiple methods and better clinical efficacy was achieved.

The results of this study show that after treatment, Da-vO_2_, OEF, SpO_2_, NO in both groups were higher than before, SjvO_2_, ERO_2_, ET, ICP, whole blood viscosity, plasma viscosity, reduced viscosity of whole blood, and hematocrit all decreased, but the changes in the observation group were greater than those in the control group, with a statistically significant difference. Mild hypothermia treatment has been used for many years in clinical practice, and it can block the process of secondary brain injury and reduce the brain metabolic rate and cerebral oxygen consumption, relieve cerebral edema, reduce intracranial pressure, improve cerebral circulation, and improve cerebral perfusion (9). However, due to the different time course, time window, rewarming speed of the treatment of mild hypothermia, there are studies (10) indicate that patients had poor results at 33°C and 35°C within 2.5 h after brain injury, but long-term hypothermia therapy for 5 d can effectively improve the humoral and cellular immune function of the patients without serious complications and mortality rate 2 weeks after operation can be reduced.

In this study, patients in the observation group were treated with an intravascular cooling device to perform intravascular heat exchange and hypothermia therapy within 6 hours after the operation, and maintaining the temperature can effectively help patients rewarming. In patients with acute cerebral infarction, hyperbaric oxygen resuscitation is more frequently used in clinical practice, which can effectively reduce the disability rate and mortality. However, the vital signs in patients with early onset are unstable and need to be placed in the ICU care treatment, and early hyperbaric oxygen therapy cannot be performed. Therefore, in this study, ventilator high-concentration oxygen therapy was performed on patients to improve the partial pressure of carotid artery and arterial oxygen, improve cerebral oxygen metabolism, and improve the prognosis of patients (11). Secondly, early percutaneous tracheotomy can also effectively improve the respiratory abnormalities caused by brain injury, correct hypoxemia, and improve prognosis. Under normal conditions, the level of ET in human blood circulation is low, but if the body is hypoxic or ischemic, collateral circulation paralysis, the vascular endothelium is damaged, secretion of ET is increased, which affects the oxygen supply and blood supply around the lesion area, resulting in collateral vessels contraction, and ET will promote the release of excitatory amino acids, accelerate neuronal death in the ischemic area, aggravate pathological changes and neurological symptoms (12). In this study, the ET of patients in the observation group was significantly reduced after comprehensive cerebral protection program after treatment, which also indicates that comprehensive brain protection program can relieve brain injury in patients with cerebral infarction.

## Conclusion

The elderly patients with acute cerebral infarction can effectively improve cerebral oxygen metabolism and vascular endothelial function, improve blood flow, which has important clinical value.

## Ethical considerations

Ethical issues (Including plagiarism, informed consent, misconduct, data fabrication and/or falsification, double publication and/or submission, redundancy, etc.) have been completely observed by the authors.

## References

[1] Sun Y Xu Y Geng L (2015). Caspase-3 inhibitor prevents the apoptosis of brain tissue in rats with acute cerebral infarction. Exp Ther Med, 10(1): 133–138. 26170924 10.3892/etm.2015.2462 PMC4487074

[2] Barrese V Taglialatela M Greenwood IA Davidson C (2015). Protective role of Kv7 channels in oxygen and glucose deprivation-induced damage in rat caudate brain slices. J Cereb Blood Flow Metab, 35(10): 1593–600. 25966943 10.1038/jcbfm.2015.83 PMC4640310

[3] Liu XT Wang W Wang LJ (2011). [Correlation of collateral circulation and prognosis in patients with acute cerebral infarction]. Zhonghua Yi Xue Za Zhi 91(11):766–8. 21600103

[4] Hu SL Huang YX Hu R Li F Feng H (2015). Osteopontin Mediates Hyperbaric Oxygen Preconditioning-Induced Neuroprotection Against Ischemic Stroke. Mol Neurobiol, 52(1): 236–43. 25146847 10.1007/s12035-014-8859-6

[5] Deng Q Zhang Y Ding H Dong Q Fu J (2015). Calcific emboli originating from the brachiocephalic trunk causing acute cerebral infarction and worm-like calcification in the right middle cerebral artery. J Clin Neurosci, 22(5): 889–90. 25769256 10.1016/j.jocn.2014.11.029

[6] Ishihara M Fujino M Ogawa H (2015). Clinical Presentation, Management and Outcome of Japanese Patients With Acute Myocardial Infarction in the Troponin Era - Japanese Registry of Acute Myocardial Infarction Diagnosed by Universal Definition (J-MINUET). Circ J, 79(6): 1255–62. 25912696 10.1253/circj.CJ-15-0217

[7] Fan AP Govindarajan ST Kinkel RP (2015). Quantitative oxygen extraction fraction from 7-Tesla MRI phase: reproducibility and application in multiple sclerosis. J Cereb Blood Flow Metab, 35(1): 131–9. 25352043 10.1038/jcbfm.2014.187 PMC4294406

[8] Talley Watts L Long JA Manga VH Huang S Shen Q Duong TQ (2015). Normobaric oxygen worsens outcome after a moderate traumatic brain injury. J Cereb Blood Flow Metab, 35(7): 1137–44. 25690469 10.1038/jcbfm.2015.18 PMC4640244

[9] Johansen FF Hasseldam H Rasmussen RS Bisgaard AS Bonfils PK Poulsen SS Hansen-Schwartz J (2014). Drug-induced hypothermia as beneficial treatment before and after cerebral ischemia. Pathobiology, 81(1): 42–52. 23989388 10.1159/000352026

[10] Sakurai T Kudo M Watanabe T (2013). Hypothermia protects against fulminant hepatitis in mice by reducing reactive oxygen species production. Dig Dis, 31(5–6): 440–6. 24281018 10.1159/000355242

[11] Hadanny A Golan H Fishlev G Bechor Y Volkov O Suzin G Ben-Jacob E Efrati S (2015). Hyperbaric oxygen can induce neuro-plasticity and improve cognitive functions of patients suffering from anoxic brain damage. Restor Neurol Neuros, 33(4):471–486. 10.3233/RNN-150517 PMC4923708 26409406

[12] Zhu M Lu M Li QJ Zhang Z Wu ZZ Li J Qian L Xu Y Wang ZY (2015). Hyperbaric oxygen suppresses hypoxic-ischemic brain damage in newborn rats. J Child Neurol, 30(1): 75–82. 24762865 10.1177/0883073814530500

